# Melatonin Supplementation and Cardiovascular Outcomes: A Systematic Review and Meta-Analysis of Randomized Trials

**DOI:** 10.3390/jcm15093444

**Published:** 2026-04-30

**Authors:** Song Peng Ang, Jia Ee Chia, Umabalan Thirupathy, Madison Laezzo, Vikash Jaiswal, Joseph Varon, Matthew Halma, Eunseuk Lee, George Davidson, Jose Iglesias

**Affiliations:** 1 Division of Cardiology, Sarver Heart Center, University of Arizona, Tucson, AZ 85724, USA; 2 Department of Medicine, Texas Tech University Health Science Center, El Paso, TX 79905, USA; 3 Cheshire Medical Center, Keene, NH 03431, USA; 4 Department of Medicine, Hackensack Meridian School of Medicine, Nutley, NJ 07110, USA; madison.laezzo@hmhn.org; 5 Endeavor Health Cardiovascular Institute, University of Chicago Pritzker School of Medicine, Chicago, IL 60026, USA; 6 Department of Medicine, University of Houston College of Medicine, Houston, TX 77004, USA; 7 Independent Medical Alliance, Washington, DC 20036, USA; 8 Open Source Medicine OÜ, Pärnu mnt. 139c, 11317 Tallinn, Estonia; 9 Department of Medicine, Rutgers Health Community Medical Center, Toms River, NJ 08755, USA; eunseuk.lee@rwjbh.org (E.L.); georgeobiidavidson@outlook.com (G.D.)

**Keywords:** melatonin, cardiovascular disease, coronary artery bypass grafting, myocardial infarction, heart failure, left ventricular ejection fraction, meta-analysis

## Abstract

**Background**: Melatonin has antioxidant and anti-inflammatory properties that may attenuate ischemia-reperfusion injury, but randomized cardiovascular trial data remain inconsistent. **Objectives**: This study sought to evaluate the association of melatonin supplementation with cardiovascular outcomes across randomized trials. **Methods**: We performed a systematic review and meta-analysis of randomized trials comparing melatonin with placebo, usual care, or no melatonin in patients with cardiovascular disease. PubMed, Embase, and CENTRAL were searched from inception to 1 January 2026. Random-effects models with Hartung–Knapp–Sidik–Jonkman confidence intervals were used. Prespecified outcomes included left ventricular ejection fraction (LVEF), change in LVEF, troponin, infarct size by cardiac magnetic resonance, heart failure outcomes, inflammatory and oxidative stress biomarkers, and adverse events. **Results**: A total of 14 randomized controlled trials involving 1027 participants were included. Melatonin significantly improved change in LVEF from baseline to follow-up (mean difference: 3.95 percentage points; 95% CI: 1.70–6.20; *p* < 0.001), with the most consistent signal in coronary artery bypass grafting studies (mean difference: 4.65 percentage points; 95% CI: 2.56–6.74). Final LVEF was numerically higher with melatonin but not statistically significant. Troponin reduction was not significant. Narrative synthesis suggested lower inflammatory and oxidative stress markers after coronary artery bypass grafting and improvement in heart failure symptoms and quality of life, whereas infarct size findings in ST-segment elevation myocardial infarction were mixed and timing-dependent. **Conclusions**: Melatonin was associated with improved LVEF change, particularly in coronary artery bypass grafting settings, but benefit was not consistently demonstrated across final LVEF, troponin, or infarct size outcomes.

## 1. Introduction

Cardiovascular disease remains the predominant driver of global mortality and morbidity worldwide, with acute coronary syndromes, heart failure, and post-surgical cardiac dysfunction representing major sources of preventable death and disability. Myocardial ischemia-reperfusion injury and maladaptive ventricular remodeling serve as critical pathophysiological determinants of long-term prognosis and functional recovery in these populations [1]. Additionally, excessive inflammatory activation and oxidative stress contribute substantially to myocardial injury and functional decline in both acute coronary syndromes and chronic heart failure, creating a therapeutic rationale for anti-inflammatory and antioxidant interventions [2,3,4,5]. Within this pathophysiological framework, melatonin has become a compelling candidate for adjunctive therapy, distinguished by properties that extend well beyond its classical role in chronobiology. Melatonin possesses potent, amphiphilic antioxidant and anti-inflammatory characteristics that theoretically mitigate the cellular cascades leading to cardiomyocyte necrosis and fibrosis [6]. Preclinical data suggest these mechanisms could arrest the progression of adverse remodeling; however, bridging the translational gap from molecular plausibility to clinical utility has proven elusive [6].

Despite a robust mechanistic basis, the existing clinical evidence regarding melatonin’s cardiovascular efficacy is fragmented and inconsistent. Randomized controlled trials aimed at preventing heart failure and improving ventricular function have reported conflicting outcomes, creating a dichotomy between successful small-scale studies and neutral larger trials [7]. Previous meta-analytical syntheses have attempted to reconcile this heterogeneity, but have been limited by small numbers of trials, varied methodological quality, and clinical heterogeneity across populations, precluding definitive conclusions regarding melatonin’s overall efficacy [7,8]. Consequently, it remains unclear whether the variance in outcomes reflects a lack of therapeutic efficacy or a failure to identify the specific clinical phenotypes and therapeutic windows in which melatonin is effective.

To address these uncertainties, we conducted a systematic review and meta-analysis of randomized controlled trials to evaluate the association between melatonin supplementation and cardiovascular outcomes. We stratified data by distinct clinical settings, including perioperative cardiac surgery, acute myocardial infarction (AMI), and heart failure with reduced ejection fraction (HFrEF). Our aim was to determine whether melatonin’s effects represent a broadly applicable cardioprotective strategy or a benefit limited to specific clinical contexts.

## 2. Methods

This systematic review and meta-analysis was conducted in accordance with the Preferred Reporting Items for Systematic Reviews and Meta-Analyses (PRISMA 2020) statement and the completed PRISMA checklist is included in Supplementary Materials. A systematic literature search was performed in PubMed, Embase, and the Cochrane Central Register of Controlled Trials (CENTRAL) from database inception through 1 January 2026. No language or date restrictions were applied. Reference lists of eligible studies and relevant review articles were also screened to identify additional studies not captured by the electronic search. The full search strategy is provided in Table S1.

### 2.1. Study Outcomes

Eligible studies were randomized controlled trials that compared melatonin with placebo, usual care, or no melatonin in patients with cardiovascular disease and reported at least 1 prespecified outcome of interest. The outcomes of interest were left ventricular ejection fraction, including final LVEF and change in LVEF from baseline to follow-up; cardiac biomarkers, including troponin; infarct size assessed by cardiac magnetic resonance imaging; heart failure-related outcomes, including New York Heart Association functional class and Minnesota Living With Heart Failure Questionnaire score; inflammatory and oxidative stress biomarkers; and adverse events. Observational studies, non-randomized interventional studies, case reports, review articles, animal studies, and duplicate reports from the same study population were excluded. These biomarkers and outcomes were selected because they represented the most commonly studied and clinically relevant endpoints across the included randomized trials, reflecting key domains of myocardial injury, ventricular performance, inflammatory and oxidative stress response, and heart failure status.

All records identified through the search were imported, and duplicates were removed using automated detection followed by manual verification. Two reviewers independently screened titles and abstracts using prespecified eligibility criteria. Full-text review of potentially eligible articles was then performed independently by the same reviewers. Disagreements at either stage were resolved through discussion and consensus, with third-reviewer adjudication when required.

### 2.2. Data Extraction

Data were extracted independently by 2 reviewers. Extracted data included study characteristics, patient population, sample size, melatonin dose and route, timing and duration of treatment, comparator, follow-up duration, and outcome data. For continuous outcomes, change-from-baseline data were used when reported. When standard deviations were not reported, they were imputed from available standard errors, 95% confidence intervals, or *p*-values using Cochrane-recommended methods. Studies with insufficient data for imputation were included in the narrative synthesis rather than the quantitative meta-analysis.

### 2.3. Risk of Bias Assessment

Risk of bias for each included trial was assessed independently by 2 reviewers using the Cochrane Risk of Bias 2 tool. This tool evaluates bias arising from the randomization process, deviations from intended interventions, missing outcome data, measurement of the outcome, and selection of the reported result. Each domain was rated as low risk of bias, some concerns, or high risk of bias, and an overall study-level judgment was assigned according to the RoB 2 algorithm. Disagreements were resolved by consensus, with adjudication by a third reviewer when necessary.

### 2.4. Statistical Analysis

Because clinical and methodological heterogeneity was anticipated across studies, all meta-analyses were performed using random-effects models, with 95% CIs estimated using the Hartung–Knapp–Sidik–Jonkman method. Continuous outcomes were pooled as mean differences when reported on a common scale and as standardized mean differences when assays or measurement scales differed. For biomarkers such as troponin, standardized mean differences were used to account for variation in assay type and reporting scale across studies. Statistical heterogeneity was assessed using the I2 statistic. Given the a priori clinical heterogeneity of the included populations, prespecified subgroup analyses were performed for the 2 principal echocardiographic outcomes including final left ventricular ejection fraction and change in left ventricular ejection fraction from baseline to follow-up, according to clinical setting: coronary artery bypass grafting, ST-segment elevation myocardial infarction treated with primary percutaneous coronary intervention, and heart failure. Subgroup analyses were performed only when sufficient data were available within a given subgroup. Accordingly, the final left ventricular ejection fraction analysis included coronary artery bypass grafting, ST-segment elevation myocardial infarction/primary percutaneous coronary intervention, and heart failure subgroups, whereas the change in left ventricular ejection fraction analysis included coronary artery bypass grafting and heart failure subgroups only. For outcomes that were not quantitatively pooled, pooling was not feasible because studies differed substantially in measurement time points, reporting formats, assay methods, outcome definitions, and follow-up intervals, precluding a methodologically sound combined estimate. These outcomes were therefore synthesized narratively. Funnel plots were examined for the principal pooled outcomes as an exploratory assessment of small-study effects.

## 3. Results

Database searching identified 508 records, of which 107 duplicates were removed. The remaining 401 records were screened, and 14 randomized controlled trials met the eligibility criteria for inclusion. Studies were excluded primarily because of wrong population, wrong outcomes, non-randomized design, or other prespecified exclusion criteria (Figure S1: PRISMA Flow Diagram).

### 3.1. Baseline Characteristics of Included Studies

A total of 14 randomized controlled trials enrolling 1027 participants were included in the final analysis, including three distinct clinical populations: coronary artery bypass grafting (CABG; seven trials), ST-elevation myocardial infarction undergoing primary percutaneous coronary intervention (STEMI/pPCI; four trials), and heart failure with reduced ejection fraction (HFrEF; three trials) (Table 1) [9,10,11,12,13,14,15,16,17,18,19,20,21,22]. The majority of trials were conducted in Iran, with the remainder from Iraq, Spain, Denmark, and Egypt. Individual trial sample sizes ranged from 34 to 130 participants. Eleven trials used a double-blind, placebo-controlled design. Melatonin doses varied considerably across trials, ranging from 3 mg administered orally at a physiologic replacement dose to 60 mg as a high-dose preoperative loading strategy, with routes including oral, sublingual, intravenous, and intracoronary administration. Timing of intervention was predominantly perioperative or post-reperfusion in the surgical and STEMI cohorts, and chronic outpatient supplementation (8 to 24 weeks) in the heart failure trials. Across the seven CABG trials, the weighted mean age ranged from approximately 52 to 65 years, the proportion of female participants was generally low (17% to 53%), and diabetes mellitus was present in 20% to 65% of patients across arms, with notable between-arm imbalances in several trials. Hypertension was prevalent across all populations, reported in 29% to 79% of participants. Baseline left ventricular ejection fraction was either not reported or reported inconsistently across trials; however, most CABG and pPCI trials required an ejection fraction exceeding 30% to 40% as an inclusion criterion, while heart failure trials specifically enrolled patients with LVEF below 40% to 50%. Background pharmacotherapy was largely uniform within CABG and HF cohorts, with the majority of patients receiving guideline-directed medical therapy including beta-blockers, statins, renin-angiotensin system inhibitors, and antiplatelet agents. No trial reported melatonin-related serious adverse events; minor adverse effects, including headache and mild gastrointestinal symptoms, were infrequently reported at supraphysiologic doses. Risk of bias across the included randomized trials was variable. Overall, four trials were judged to be at low risk of bias, seven raised some concerns, and three were rated at high risk of bias (Figures S2 and S3). The most frequent concerns related to the randomization process, particularly incomplete reporting of allocation concealment, while deviations from intended interventions were more relevant in open-label studies. Missing outcome data was an important driver of high-risk judgments in trials with substantial attrition or incomplete imaging follow-up, whereas outcome measurement was generally less problematic because several studies used standardized laboratory, echocardiographic, or CMR-based assessments. Concerns regarding selection of the reported result were also present in smaller trials with multiple biomarker endpoints or time points without clearly documented prespecification. Overall, these findings indicate that the pooled estimates should be interpreted with appropriate caution, particularly where apparent benefit was driven by small trials with methodological limitations.

### 3.2. Final LVEF and Change in LVEF from Baseline to Follow-Up

**Figure 1:**
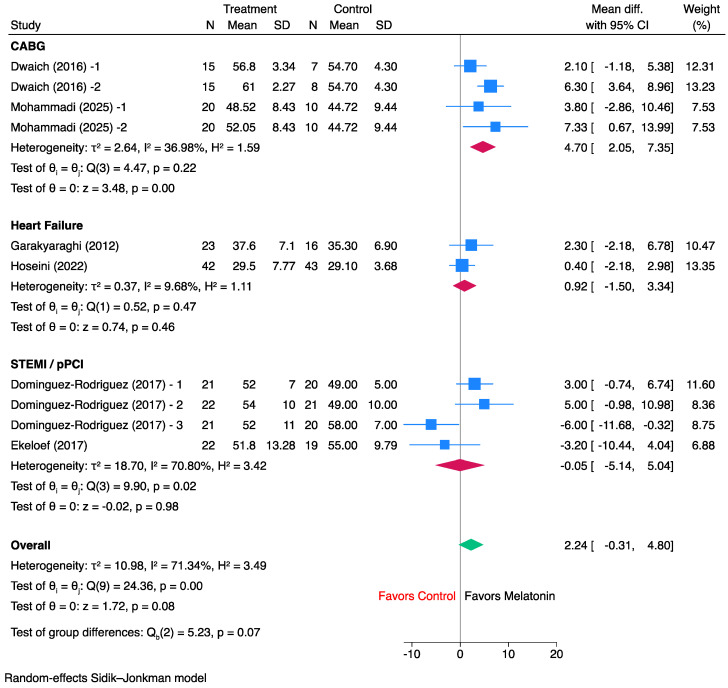
Forest plot of the effect of melatonin versus control on final left ventricular ejection fraction.

Across all available randomized controlled trials, pooled analysis of final left ventricular ejection fraction (LVEF) showed that melatonin was associated with a numerically higher final LVEF than control, although the overall difference did not reach statistical significance (mean difference, 2.24 percentage points; 95% CI, −0.31 to 4.80; *p* = 0.08) (Figure 1). Heterogeneity was considerable across the included comparisons (τ^2^ = 10.98; I^2^ = 71.34%; Q = 24.36), indicating substantial between-study variability in effect estimates. Directionally, the benefit appeared more consistent in the CABG trials, whereas results from STEMI/pPCI and chronic heart failure studies were more variable, contributing to the neutral overall pooled estimate. Leave-one-out sensitivity analysis showed that the pooled estimate for final LVEF remained consistently in favor of melatonin, with mean differences ranging from 1.63 to 3.03 percentage points (Figure S4). Exclusion of most individual studies did not materially alter the overall interpretation; however, omission of Dominguez-Rodriguez et al. (3rd tertile) and Ekeloef et al. rendered the pooled effect statistically significant, suggesting that the overall result was directionally robust but of borderline statistical stability.

**Figure 2:**
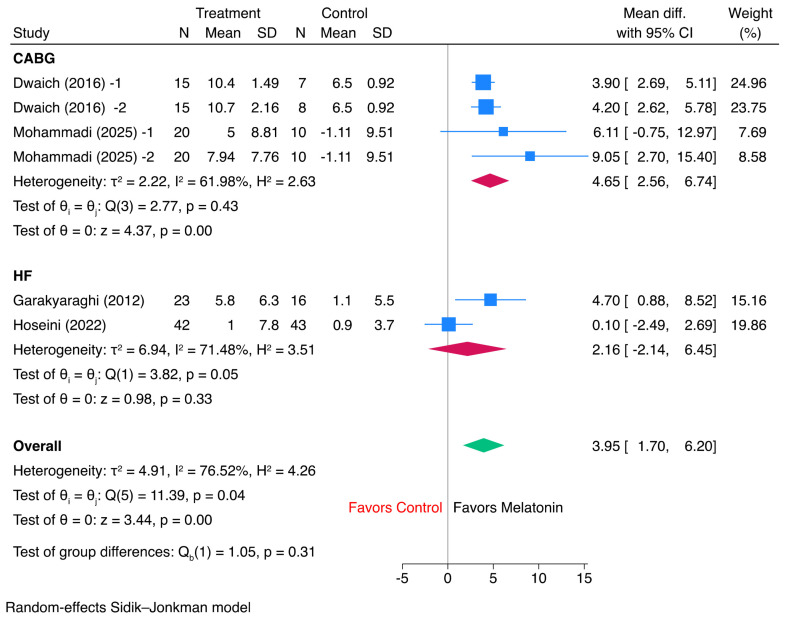
Forest plot of the effect of melatonin versus control on change in left ventricular ejection fraction.

For change in LVEF, pooled analysis showed that melatonin significantly improved LVEF compared with control, with an overall mean difference of 3.95 percentage points (95% CI, 1.70 to 6.20; *p* < 0.001) (Figure 2). Between-study heterogeneity was substantial (I^2^ = 76.52%, τ^2^ = 4.91). In subgroup analysis, the effect was significant among CABG studies (mean difference, 4.65; 95% CI, 2.56 to 6.74; *p* < 0.001), whereas the heart failure subgroup showed a numerically favorable but non-significant effect (mean difference, 2.16; 95% CI, −2.14 to 6.45; *p* = 0.33). There was no significant difference between subgroups (Qb = 1.05, *p* = 0.31), suggesting that the overall benefit in LVEF change was not statistically modified by study population. Sensitivity analysis using leave-one-out method showed that effect size remained consistent and unaltered in terms of direction, with MD ranges from 3.4 to 4.5 (Figure S5).

### 3.3. Change in Troponin from Baseline to Follow-Up

**Figure 3:**
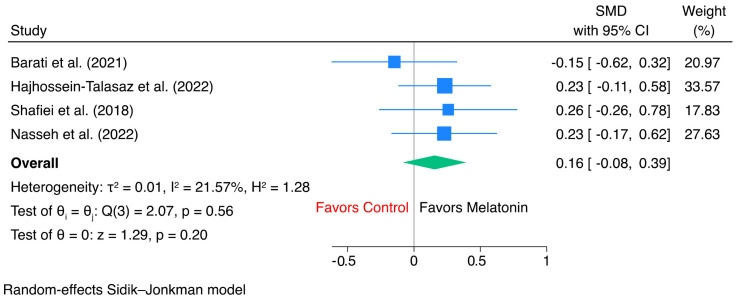
Forest plot of the effect of melatonin versus control on troponin levels (CABG population only).

For troponin, pooled analysis of four randomized controlled trials showed that melatonin was associated with a numerically lower troponin burden than control, although the overall effect was not statistically significant (SMD, 0.16; 95% CI, −0.08 to 0.39; *p* = 0.20) (Figure 3) [11,12,13,14]. Between-study heterogeneity was low (I^2^ = 21.6%; τ^2^ = 0.01; Q = 2.07, *p* = 0.56), indicating generally consistent findings across trials. Individual study estimates were mostly in favor of melatonin, with effect sizes ranging from −0.15 to 0.26, and the largest weight was contributed by Hajhossein-Talasaz et al. [13] (33.6%). In leave-one-out sensitivity analysis, the pooled effect remained directionally favorable to melatonin across all iterations (SMD range, 0.12 to 0.24); omission of Barati et al. [12] resulted in a borderline statistically significant pooled estimate (SMD, 0.24; 95% CI, 0.00 to 0.47; *p* = 0.046), whereas omission of the other trials did not materially change the overall interpretation (Figure S6).

### 3.4. Anti-Inflammatory Markers

Across the three included trials, melatonin was associated with consistent attenuation of oxidative stress and inflammatory biomarkers after CABG, although the magnitude and temporal pattern of effect varied across studies. Overall, melatonin was linked to lower malondialdehyde (MDA) and tumor necrosis factor-α (TNF-α) levels compared with placebo or control. Shafiei et al. [11] demonstrated significantly lower perioperative MDA and TNF-α levels with preoperative melatonin, while Mohammadi et al. [15] reported significant reductions in both biomarkers at 60-day follow-up with postoperative melatonin, without a clear dose–response difference between the 5 mg and 10 mg regimens. Casper et al. [9] similarly found lower TNF-α levels with high-dose preoperative melatonin, with the effect appearing most prominent in the early perioperative period and diminishing by 24 h. These outcomes were not quantitatively pooled because the studies reported the biomarkers at different time points, used different dosing and administration protocols, and did not provide sufficiently comparable outcome data for meta-analysis.

### 3.5. Heart Failure Outcomes

Across the heart failure trials, melatonin showed a generally favorable but somewhat heterogeneous signal for patient-centered outcomes. In the MeHR trial of stable HFrEF, 24 weeks of melatonin 10 mg nightly improved health-related quality of life, with a treatment effect favoring melatonin on MLHFQ score, and was also associated with better NYHA class at study end. In the earlier Garakyaraghi trial [20], melatonin 3 mg for 2 months likewise improved functional status, with NYHA class improving only in the melatonin group and not in controls, supporting a possible symptomatic benefit. By contrast, in the cardiac cachexia trial by Jafari-Vayghan et al. [22], quality-of-life improvement appeared to be driven mainly by the BCAA-containing regimens, whereas melatonin alone showed more modest benefit, and NYHA class was used primarily as a matching characteristic rather than reported as a main efficacy outcome. These outcomes were not quantitatively pooled because functional status and quality-of-life endpoints were reported heterogeneously across studies, with variability in outcome definitions, instruments, co-interventions, and statistical presentation.

### 3.6. Infarct Size in STEMI Population

For CMR-assessed infarct size in STEMI, the evidence was mixed and appeared to be strongly influenced by timing of administration. In the IMPACT trial [18], where patients received intracoronary plus intravenous melatonin during pPCI and underwent CMR at day 4 (±1 day), infarct size was not significantly different between groups; infarct size as a proportion of left ventricular mass was 12.7% (95% CI 8.3–19.2) in the melatonin group versus 10.7% (95% CI 8.5–13.6) in the placebo group, and no secondary CMR parameter differed significantly overall, consistent with a neutral effect on reperfusion injury. In contrast, a post hoc analysis of the MARIA trial [17] suggested a time-dependent effect; although the parent trial had reportedly failed to reduce infarct size overall, stratification by symptom-onset-to-balloon time showed a significant interaction, with smaller infarct size among melatonin-treated patients in the earliest tertile (14.6 ± 14.2% vs. 24.9 ± 9.0%; *p* = 0.003), no clear difference in the intermediate tertile (22 ± 11% vs. 20 ± 11%; *p* = 0.530), and larger infarct size with melatonin in the latest tertile (20.5 ± 8.7% vs. 11.2 ± 5.2%; *p* = 0.001). Overall, these findings suggest that melatonin does not consistently reduce infarct size by CMR after STEMI, though a possible benefit with very early administration remains hypothesis-generating.

### 3.7. Publication Bias

Visual inspection of funnel plots for the outcomes including final LVEF and change in LVEF from baseline to follow-up did not suggest marked asymmetry (Figures S7 and S8). However, given the small number of studies available for each pooled analysis, the ability of funnel plots to detect small-study effects or publication bias was limited, and these findings should be interpreted cautiously.

## 4. Discussion

In this study, melatonin supplementation was associated with a signal toward preservation of left ventricular systolic function, although the magnitude and consistency of benefit differed across clinical settings. The strongest quantitative finding was observed for change in LVEF from baseline to follow-up, whereas the pooled difference in final LVEF was not statistically significant. Similarly, change in troponin was not significantly reduced overall, despite directional improvement in inflammatory and oxidative stress markers in several perioperative studies. Narrative findings from heart failure trials suggested potential benefit in quality of life and functional capacity, whereas results in STEMI were less consistent and appeared to depend on the timing of administration in relation to reperfusion. The pooled mean difference in change in LVEF was +3.95 percentage points (95% CI 1.70–6.20), with the effect driven largely by the CABG subgroup (+4.65 percentage points; 95% CI 2.56–6.74). However, the certainty of evidence for the primary echocardiographic outcomes was low because of indirectness related to heterogeneous populations, imprecision from small sample sizes and wide confidence intervals, and risk of bias in several included trials [23,24]. Collectively, these findings support a context-dependent cardioprotective effect of melatonin rather than a uniform benefit across cardiovascular conditions.

The most consistent signal was observed in patients undergoing CABG, among whom melatonin was associated with greater improvement in LVEF and attenuation of inflammatory and oxidative stress markers. This pattern is biologically plausible. Cardiac surgery represents a relatively controlled ischemia-reperfusion setting in which the timing of injury is known, the inflammatory response is pronounced, and treatment can be administered in close temporal proximity to the inciting event [25,26]. Under these conditions, an intervention with antioxidant and anti-inflammatory properties may be more likely to achieve meaningful myocardial exposure during the relevant therapeutic window [9]. Nevertheless, these findings should be interpreted cautiously given the modest sample sizes, heterogeneity in dose and route of administration, and variability in outcome ascertainment across trials. Regimens ranged from 3 mg orally to 60 mg given preoperatively, delivered by oral, sublingual, or intravenous routes, raising the likelihood of materially different biologic exposure across studies. Moreover, several CABG trials were judged to have some concerns or high risk of bias, particularly because of incomplete allocation concealment and baseline imbalances. Accordingly, the apparent signal in the CABG subgroup should be interpreted as hypothesis-supporting rather than definitive.

By contrast, the evidence in STEMI was less consistent. CMR-assessed infarct size did not demonstrate a reproducible overall reduction with melatonin, and available data suggest that any potential benefit may depend critically on very early administration before or at the time of reperfusion [24]. This pattern is biologically plausible, because melatonin is hypothesized to exert benefit primarily through attenuation of reperfusion-related oxidative stress, mitochondrial permeability transition, and downstream microvascular injury, processes that evolve rapidly in the earliest phase after coronary reopening. The signal observed in the earliest treatment window, contrasted with neutral or unfavorable findings when treatment was effectively later relative to ischemic duration, is therefore consistent with a narrow therapeutic window rather than a uniformly effective reperfusion adjunct. Taken together, the mixed findings across AMI trials therefore do not support melatonin as a broadly effective therapy across all infarct presentations, but instead suggest that any benefit, if present, is likely to depend on precise timing, route, and dose selection [16,17,18,19].

Evidence in chronic HFrEF remains preliminary. The available trials were few, small, and clinically heterogeneous, with substantial differences in baseline phenotype, treatment duration, cointerventions, and outcome assessment [23]. While some studies suggested improvement in MLHFQ score and NYHA functional class, these signals were not consistent, and the current evidence does not support firm conclusions regarding symptomatic or structural benefit. Any effect of melatonin in chronic heart failure may be smaller than in acute or peri-injury settings, or limited to selected phenotypes characterized by increased oxidative stress, inflammation, or neurohormonal activation. This pattern is also mechanistically plausible. Unlike perioperative ischemia-reperfusion or acute infarction, chronic heart failure reflects a sustained syndrome of neurohormonal activation, ventricular remodeling, systemic inflammation, and comorbidity burden, such that any antioxidant or anti-inflammatory effect of melatonin would be expected to be more modest, slower to emerge, and less likely to translate into measurable structural improvement over the relatively short follow-up periods of the available trials. Accordingly, the observed signal in HFrEF may be more consistent with a limited adjunctive effect on symptoms or biomarker profiles than with a robust reverse-remodeling effect. No melatonin-related serious adverse events were reported across the trials. Minor adverse effects, primarily headache and mild gastrointestinal symptoms, were infrequent and occurred mainly at supraphysiologic doses. Nonetheless, short follow-up and inconsistent safety reporting preclude definitive conclusions regarding long-term tolerability, especially at higher doses or with parenteral administration. Even so, the absence of major safety signals supports continued clinical investigation in cardiovascular populations.

## 5. Limitations

Several limitations should be considered. First, the total number of randomized trials was small, and most studies enrolled limited numbers of participants, increasing susceptibility to imprecision and small-study effects. Second, there was substantial clinical and methodological heterogeneity across trials, including differences in patient population, melatonin formulation, dose, route, duration, and timing of administration. Third, outcome reporting was inconsistent, with some studies reporting final values, others reporting change scores, and several clinically relevant endpoints available only for narrative synthesis, thereby limiting quantitative pooling. Fourth, follow-up duration was generally short, precluding assessment of whether observed physiologic changes translate into differences in heart failure events, recurrent ischemic events, or mortality. Fifth, adverse event reporting was inconsistent across studies. Although funnel plots were examined, formal assessment of publication bias is inherently limited when only a small number of studies are available and should not be interpreted as evidence that such bias is absent. Sixth, we acknowledged that this systematic review was not prospectively registered in PROSPERO. Seventh, the variability in melatonin dosing (3 mg to 60 mg), route of administration (oral, sublingual, intravenous, intra-coronary), and intervention timing across trials represents a pharmacologically important limitation given that melatonin’s proposed mechanisms are highly dose- and time-dependent; the pooled estimates should not be interpreted as characterizing the effect of any single melatonin regimen. Eighth, the primary pooled positive finding which is the improvement in change in LVEF should be interpreted with particular caution, as change-from-baseline endpoints are inherently more susceptible to regression to the mean and baseline imbalances than final absolute values.

## 6. Future Directions

These findings have implications for future trial design. Rather than evaluating melatonin across broadly heterogeneous cardiovascular populations, future trials should focus on narrowly defined clinical settings in which the proposed mechanism is most likely to be relevant, particularly perioperative cardiac surgery and acute reperfusion scenarios with protocolized early administration. Studies should standardize dose, route, and timing of therapy; prespecify clinically meaningful endpoints; incorporate imaging and biomarker-based assessments where appropriate; and ensure adequate follow-up to determine whether short-term physiologic effects translate into durable clinical benefit.

## 7. Conclusions

In this study, melatonin was associated with improvement in change in left ventricular ejection fraction, with the most consistent signal observed in perioperative CABG settings. However, benefit was not consistently demonstrated across final LVEF, troponin, or CMR-assessed infarct-size outcomes, and treatment effects may vary according to clinical context, timing of administration, and dosing strategy. These findings support the possibility of a context-specific cardioprotective effect rather than a uniform benefit across cardiovascular syndromes. Larger, methodologically rigorous randomized trials with standardized dosing, route, timing, and prespecified clinical and imaging end points are needed before melatonin can be considered for routine cardiovascular use.

## Figures and Tables

**Table 1:** Baseline characteristics of included studies.

CABG							
Study	Year	Study Population	N	Intervention	Control	Age, y	Sex
Dwaich et al. [10]	2016	Ischemic heart disease undergoing elective CABG	45	Melatonin 10 mg/day or 20 mg/day orally for 5 d pre-op (n = 15 each)	Placebo for 5 d pre-op (n = 15)	52.3/53.9/52.5 (Mel 10/Mel 20/control); range 45–65	13/2/11/4/12/3 (M/F; Mel 10/Mel 20/control)
Shafiei et al. [11]	2018	Elective CABG, ischemic heart disease; LVEF ≥ 40%	88	Melatonin 5 mg orally TID from 24 h pre-op + 1 dose 1 h pre-op (n = 30); separate NAC arm (n = 28)	Placebo (n = 30)	62.0 ± 8.8/57.7 ± 11.2/61.6 ± 7.7 (melatonin/NAC/control); range 39–76	15/15/18/10/14/16 (M/F; melatonin/NAC/control)
Barati et al. [12]	2020	Adults 18–70 y undergoing elective on-pump CABG	70	Melatonin 12 mg SL evening before + 12 mg 1 h pre-op (n = 35)	Standard care/no melatonin (n = 35)	64 (58–68) vs. 65 (62–68), median (melatonin vs. control)	26/9 vs. 29/6 (M/F; melatonin vs. control)
Hajhossein-Talasaz et al. [13]	2022	Elective on-pump CABG	130	Melatonin 3 mg orally from 3 d pre-op to discharge (n = 65)	Standard care/no melatonin (n = 65)	59.9 ± 9.6 vs. 60.8 ± 8.0 (melatonin vs. control)	47/18 vs. 45/20 (M/F; melatonin vs. control)
Nasseh et al. [14]	2022	Adults 50–80 y undergoing elective CABG (ASA II–III)	100	Melatonin 3 mg night before surgery, morning of surgery, and nightly for 3 d post-op (n = 50)	Placebo on the same schedule (n = 50)	62.8 ± 9.2 vs. 60.2 ± 8.6 (melatonin vs. control)	37/13 vs. 34/16 (M/F; melatonin vs. control)
Casper et al. [9]	2025	Adults undergoing on-pump CABG	34	Melatonin 60 mg/day orally for 5 d pre-op (n = 17)	Placebo for 5 d pre-op (n = 17)	56.6 ± 8.0 vs. 51.8 ± 7.8 (melatonin vs. placebo)	14/3 vs. 12/5 (M/F; melatonin vs. placebo)
Mohammadi et al. [15]	2025	Adults 30–70 y after elective CABG	52	Melatonin 5 mg or 10 mg nightly for 60 d starting 8–10 d post-op (n = 17 each)	Placebo nightly for 60 d (n = 18)	64.47 ± 7.91/61.76 ± 8.99/60.28 ± 7.36 (Mel 5/Mel 10/control)	10/7/12/5/11/7 (M/F; Mel 5/Mel 10/control)
STEMI/PCI							
Study	Year	Study population	N	Intervention	Control	Age, y	Sex
Ghaeli et al. [16]	2015	STEMI undergoing primary PCI	40	Melatonin 3 mg orally during hospitalization (n = 20)	Standard therapy only (n = 20)	58.64 ± 12.91 vs. 58.13 ± 11.87 (melatonin vs. control)	15/5 vs. 15/5 (M/F; melatonin vs. control)
Dominguez-Rodriguez et al. [17]	2017	First STEMI within 6 h undergoing primary PCI	125	IV melatonin 51.7 μmol over 60 min pre-pPCI + IC melatonin 8.6 μmol after reperfusion (n = 63)	Matching placebo during pPCI (n = 62)	n/a (reported by symptom-to-balloon tertile only)	n/a (reported by symptom-to-balloon tertile only)
Ekeloef et al. [18]	2017	STEMI within 6 h, TIMI 0–1 occlusion, primary PCI	48	IC melatonin 1 mg at reperfusion + IV melatonin 50 mg over 6 h (n = 24)	Matching placebo saline (n = 24)	61.7 (95% CI 56.2–66.9) vs. 64.0 (59.4–68.7)	20/4 vs. 18/6 (M/F; melatonin vs. control)
Dominguez-Rodriguez et al. [19]	2022	STEMI undergoing successful primary PCI within 3 h of symptom onset	94	IV melatonin 12 mg over 60 min immediately pre-PCI (n = 45)	Placebo saline over 60 min immediately pre-PCI (n = 49)	60.4 (57.0–77.3) vs. 60.5 (54.9–69.7), median	28/17 vs. 23/26 (M/F; melatonin vs. control)
HF							
Study	Year	Study population	N	Intervention	Control	Age, y	Sex
Garakyaraghi et al. [20]	2012	Stable HF (NYHA II–III, LVEF < 50%)	39	Melatonin 3 mg nightly for 2 mo (n = 23)	Placebo nightly for 2 mo (n = 16)	63.6 ± 6.6 vs. 65.8 ± 12.5 (melatonin vs. control)	17/6 vs. 10/6 (M/F; melatonin vs. control)
Hoseini et al. [21]	2022	Stable HFrEF (NYHA II–III, LVEF < 40%)	92	Melatonin 10 mg nightly for 24 wk (n = 46)	Placebo nightly for 24 wk (n = 46)	63.5 (56.7–70.2) vs. 58.5 (54.0–67.2), median	40/6 vs. 40/6 (M/F; melatonin vs. control)
Jafari-Vayghan et al. [22]	2022	Cardiac cachexia with chronic HF	70	Melatonin 20 mg/day (n = 18); separate BCAA arm (n = 17) and melatonin + BCAA arm (n = 18) for 8 wk	Placebo/cornstarch comparator (n = 17)	55.78 ± 11.57/50.18 ± 11.78/46.28 ± 14.22/50.82 ± 11.22 (Mel/BCAA/Mel + BCAA/control)	13/5/12/5/13/5/12/5 (M/F; Mel/BCAA/Mel + BCAA/control)

## Data Availability

All data underlying this study are available within the article and its Supplementary Materials.

## Supplementary Materials

The following supporting information can be downloaded at https://www.mdpi.com/article/10.3390/jcm15093444/s1, Table S1, Search Strategy. Figure S1, PRISMA Flow Diagram. Figure S2, Risk of bias for included randomized trials using the Cochrane RoB 2 tool. Figure S3, Summary of overall risk of bias. Figure S4, Leave-one-out sensitivity analysis for final left ventricular ejection fraction. Figure S5, Leave-one-out sensitivity analysis for change in left ventricular ejection fraction. Figure S6, Leave-one-out sensitivity analysis for troponin levels. Figure S7, Funnel plot for final left ventricular ejection fraction. Figure S8, Funnel plot for change in left ventricular ejection fraction. PRISMA 2020 checklist [27].
