# Supplementary material for: Melatonin Supplementation and Cardiovascular Outcomes: A Systematic Review and Meta-Analysis of Randomized Trials
Source: J Clin Med. 2026 Apr 30;15(9):3444. doi: 10.3390/jcm15093444 (PMC13164420; doi:10.3390/jcm15093444)
Supplement: Supplementary file 1 [file jcm-15-03444-s001.zip › Supplementary Materials- melatonin.pdf]

Supplementary Materials

Table S1 Search Strategy
Figure S1 PRISMA Flow Diagram
Figure S2 Risk of bias for included randomized trials using the Cochrane RoB 2 tool.
Figure S3 Summary of overall risk of bias
Figure S4 Leave-one-out sensitivity analysis for final left ventricular ejection fraction
Figure S5 Leave-one-out sensitivity analysis for change in left ventricular ejection fraction
Figure S6 Leave-one-out sensitivity analysis for troponin levels
Figure S7 Funnel plot for final left ventricular ejection fraction
Figure S8 Funnel plot for change in left ventricular ejection fraction

Table S1 Search Strategy

Database	Search terms
PubMed	("melatonin"[MeSH Terms] OR "melatonin"[tiab] OR "N-acetyl-5-methoxytryptamine"[tiab])<br>AND<br>("coronary artery bypass"[MeSH Terms] OR "CABG"[tiab] OR "cardiac surgery"[tiab] OR "myocardial infarction"[MeSH Terms] OR "ST elevation myocardial infarction"[tiab] OR "STEMI"[tiab] OR "percutaneous coronary intervention"[MeSH Terms] OR "primary PCI"[tiab] OR "heart failure"[MeSH Terms] OR "cardiac failure"[tiab] OR "coronary artery disease"[MeSH Terms] OR "ischemic heart disease"[tiab] OR "reperfusion injury"[MeSH Terms] OR "ischemia-reperfusion"[tiab])<br>AND<br>(randomized controlled trial[pt] OR controlled clinical trial[pt] OR "randomized"[tiab] OR "randomised"[tiab] OR "randomly allocated"[tiab] OR "placebo"[tiab])
Embase	('melatonin'/exp OR melatonin:ti,ab OR 'n-acetyl-5-methoxytryptamine':ti,ab) AND ('coronary artery bypass'/exp OR cabg:ti,ab OR 'cardiac surgery'/exp OR 'cardiac surgery':ti,ab OR 'myocardial infarction'/exp OR 'myocardial infarction':ti,ab OR 'st elevation myocardial infarction':ti,ab OR stemi:ti,ab OR 'percutaneous coronary intervention'/exp OR 'primary pci':ti,ab OR 'heart failure'/exp OR 'cardiac failure':ti,ab OR 'coronary artery disease'/exp OR 'ischemic heart disease':ti,ab OR 'reperfusion injury'/exp OR 'ischemia-reperfusion':ti,ab) AND ('randomized controlled trial'/exp OR 'controlled clinical trial'/exp OR randomized:ti,ab OR randomised:ti,ab OR 'randomly allocated':ti,ab OR placebo:ti,ab)
Cochrane Library	#1 melatonin OR "N-acetyl-5-methoxytryptamine"<br><br>#2 "coronary artery bypass" OR CABG OR "cardiac surgery"<br>OR "myocardial infarction" OR STEMI OR "ST elevation myocardial infarction"<br>OR "percutaneous coronary intervention" OR "primary PCI"<br>OR "heart failure" OR "cardiac failure" OR "coronary artery disease"<br>OR "ischemic heart disease" OR "reperfusion injury" OR "ischemia-reperfusion"<br><br>#3 #1 AND #2

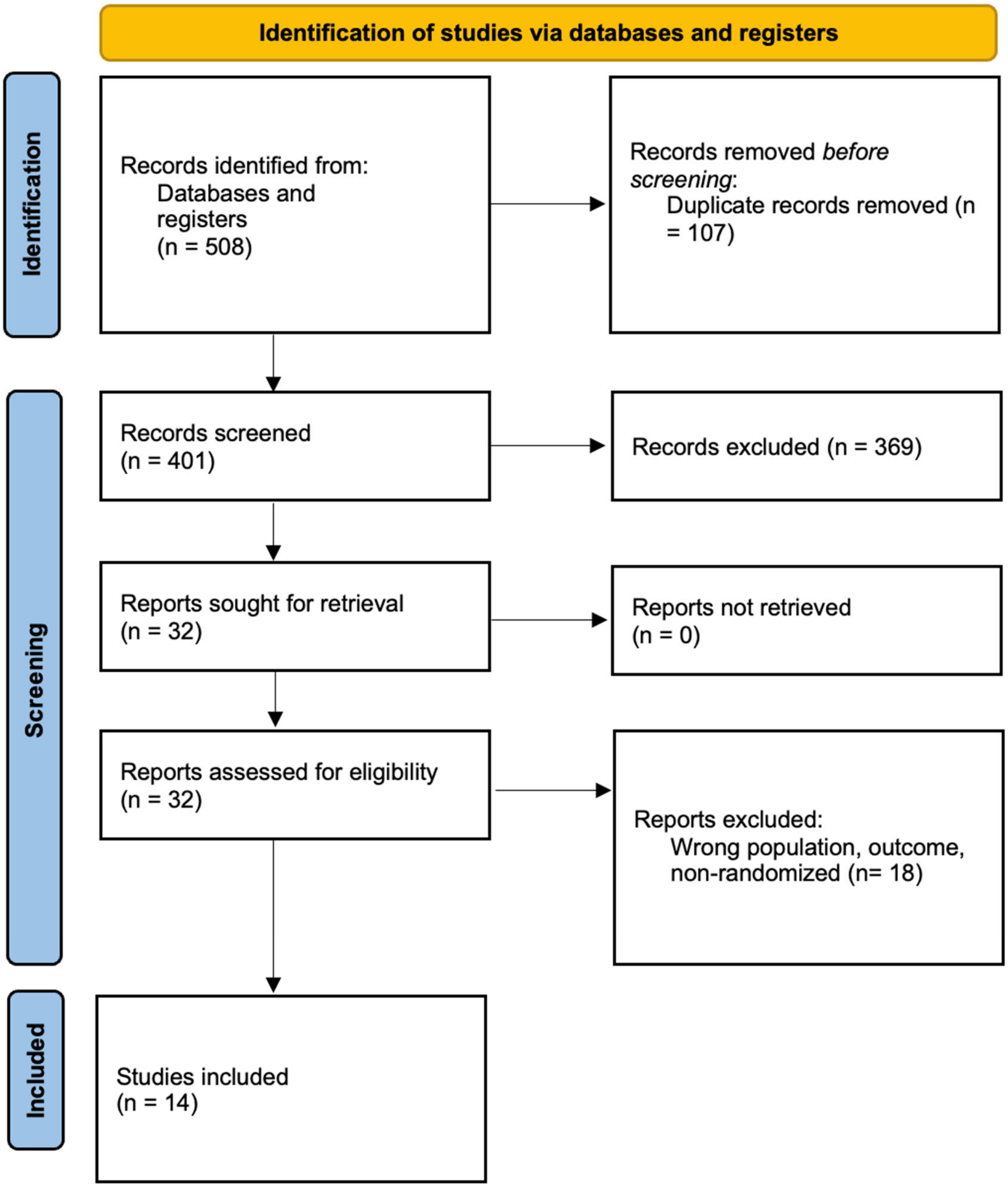

Figure S1 PRISMA Flow Diagram

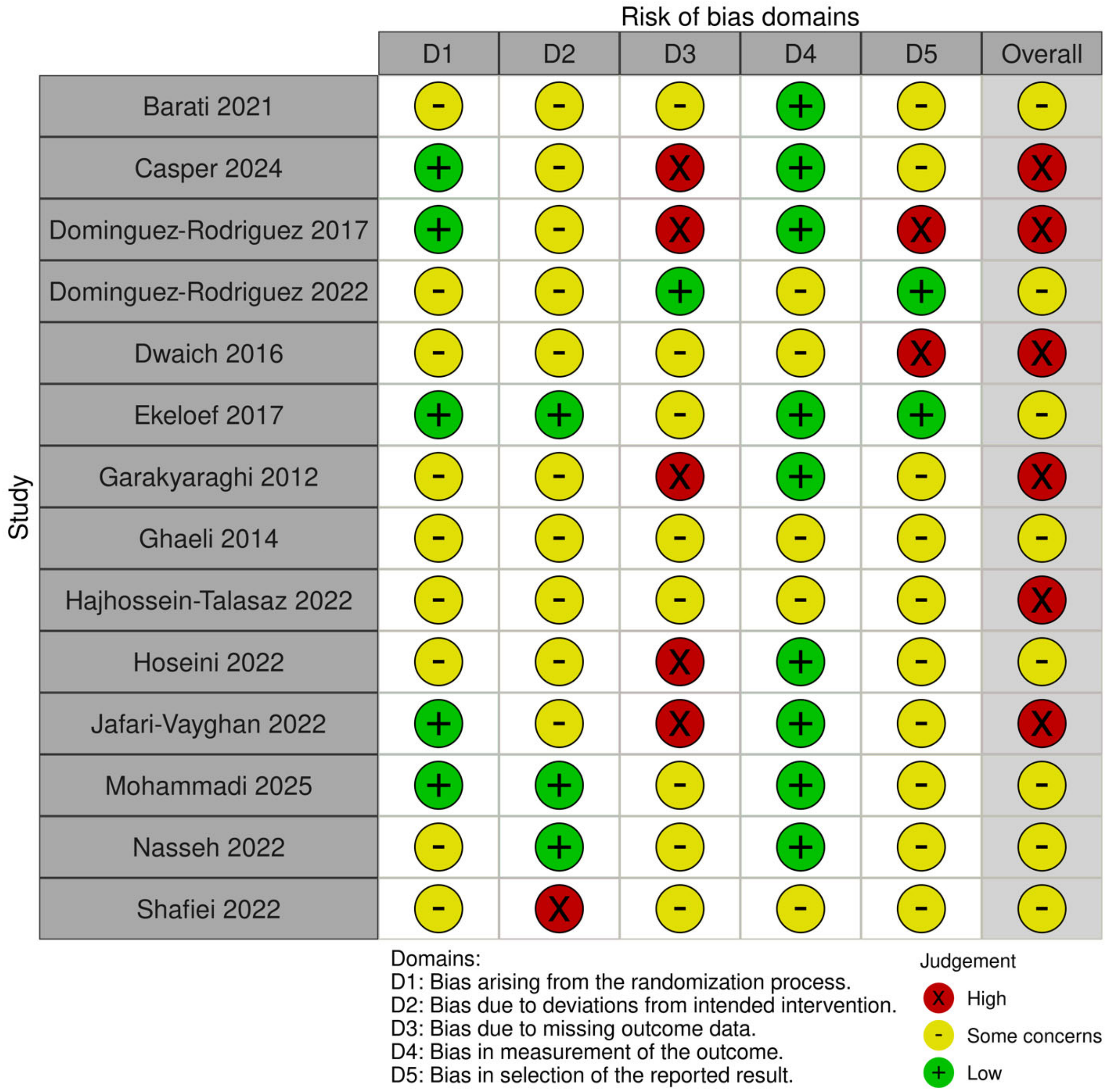

Figure S2 Risk of bias for included randomized trials using the Cochrane RoB 2 tool

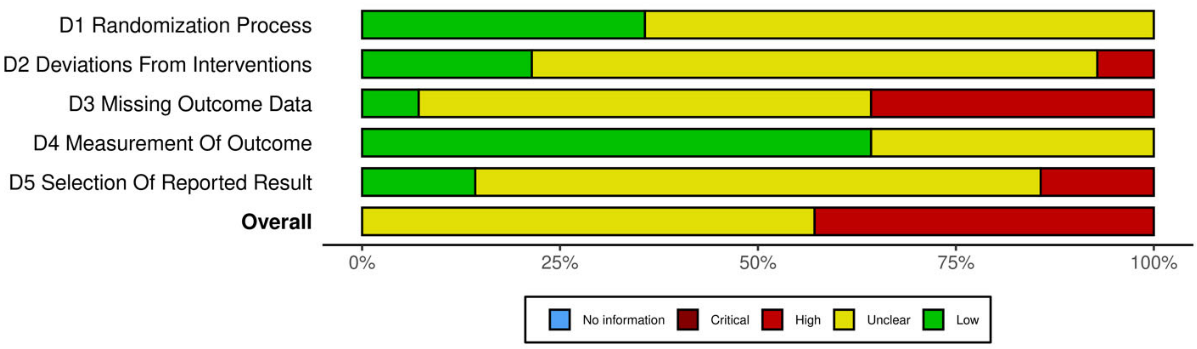

Figure S3 Summary of overall risk of bias

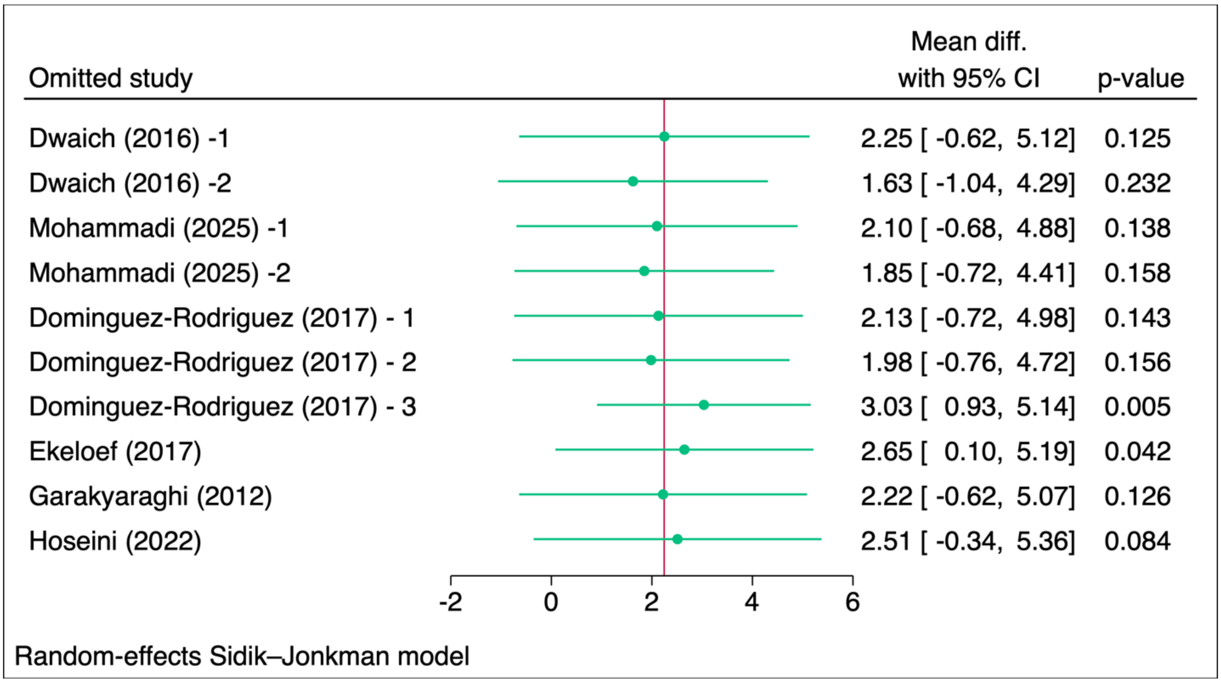

Figure S4 Leave-one-out sensitivity analysis for final left ventricular ejection fraction

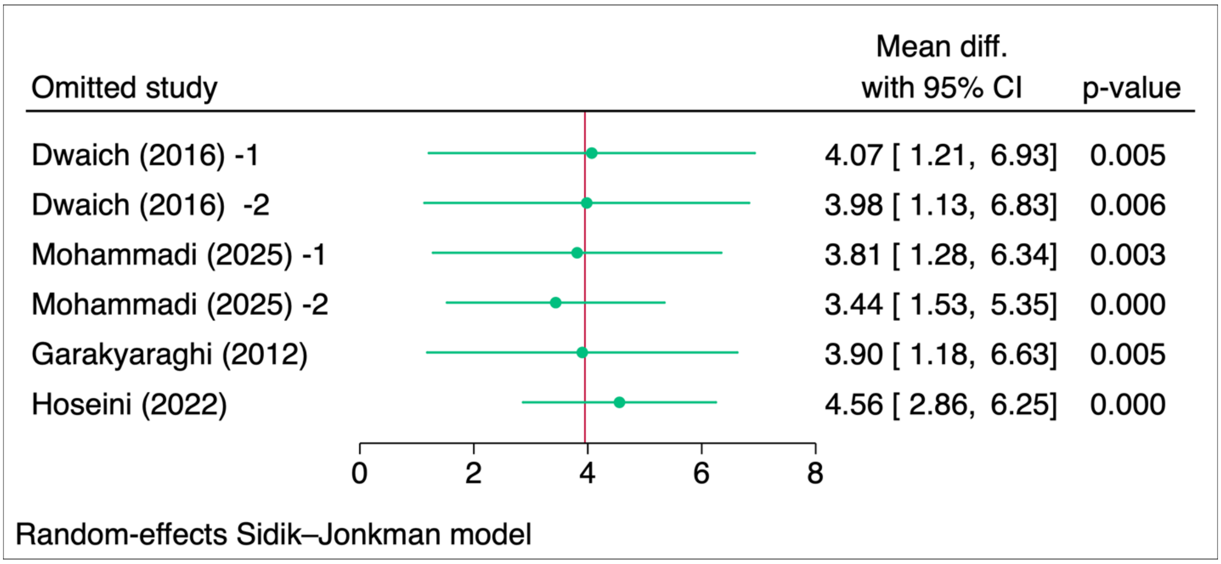

Figure S5 Leave-one-out sensitivity analysis for change in left ventricular ejection fraction

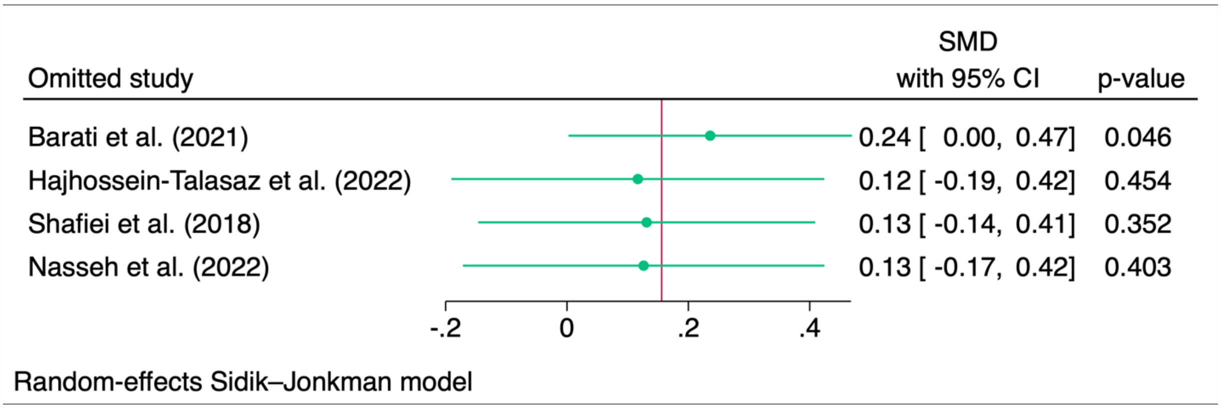

Figure S6 Leave-one-out sensitivity analysis for troponin levels

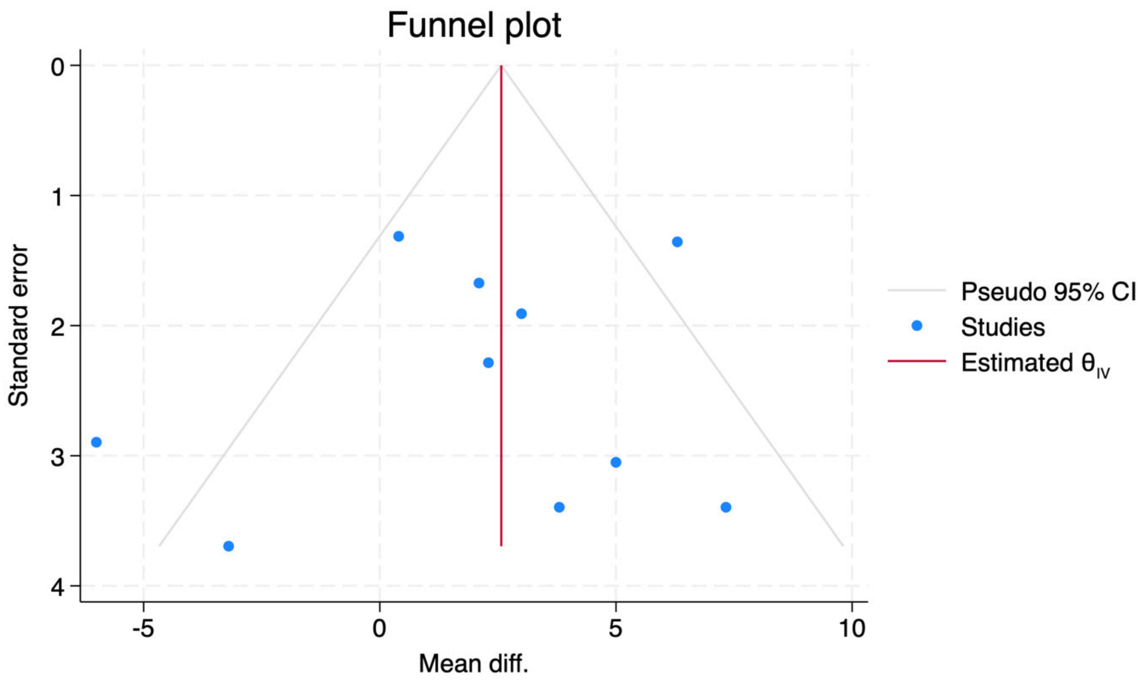

Figure S7 Funnel plot for final left ventricular ejection fraction

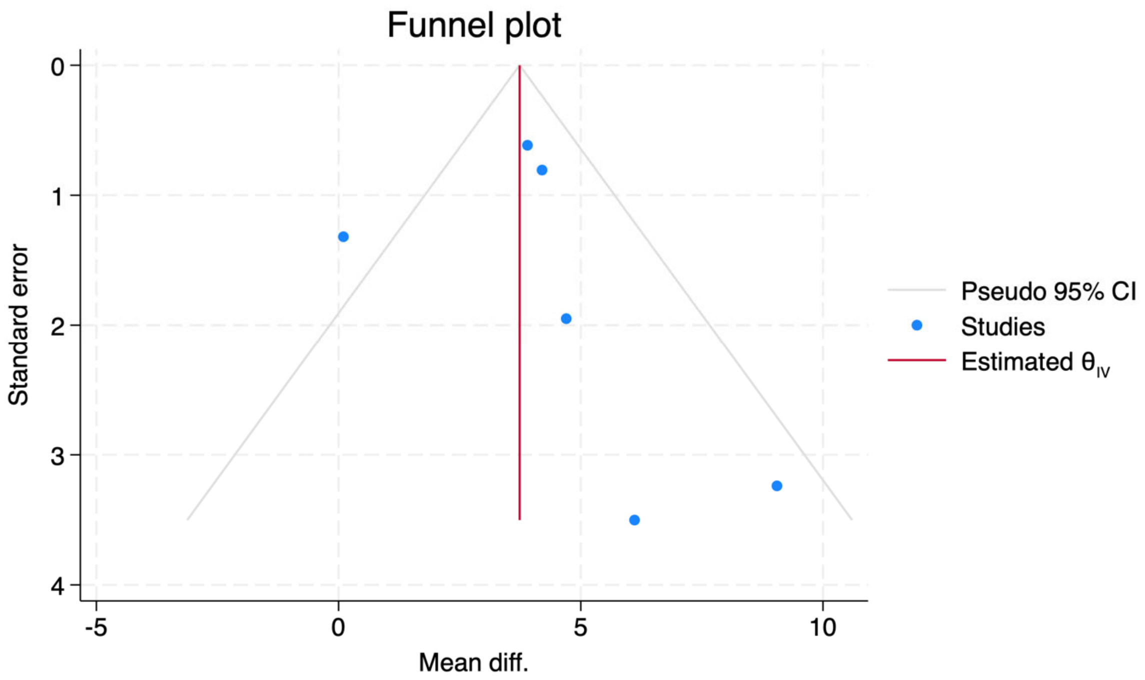

Figure S8 Funnel plot for change in left ventricular ejection fraction
